# What the Very Poor Eat—Dietaries of Slums in American Cities Studied*Special Washington Correspondence of the Boston Transcript.

**Published:** 1899-02

**Authors:** 


### WHAT THE VERY POOR EAT—DIETARIES OF SLUMS IN AMERICAN CITIES STUDIED.*

Experts of the Department of Agriculture have been making a special study of the foods eaten by foreign-born people in American cities. Their attention has been devoted chiefly to the very poor, because with them the problem of food-supply is one of acute and even overwhelming importance. The well-to-do citizen regards the supplies for his table as a mere incidental, the bulk of his income being expended in other ways, and largely for luxuries. But with the poor it is quite otherwise; mere maintenance for their bodies is the chief anxiety, and absorbs the bulk of the money product of the family.

The experts made their studies in districts of various cities where people of many nationalities have their homes. As might be imagined, they encountered many difficulties. Not a few of these aliens, settled on American soil, were suspicious of the motives of the persons who desired to subject them to a quasi-microscopic observation in regard to their eating and drinking. Naturally they could not understand it, and imagined that the agents of the Department of Agriculture were spies set to watch their private affairs. In several instances it was necessary to pay them for the privilege of permitting the dietary studies to be made.

Now, the method of the dietary study is simple enough. When a family is under this kind of observation, two visits are made to it daily, and on each occasion all food-materials bought since the previous call are carefully weighed. The family is instructed to weigh portions of flour and sugar, if considerable quantities of these are on hand, and to use only from the weighed portions. Of course there are chances of error. Things run out just before meal-time, and the smallest girl is dispatched with orders to purchase five cents' worth of tea, three cents' worth of crackers, or what not. But in most instances pretty definite data were secured.

The experts had their own figures as to the per cent. of muscle-forming substance in a pound of beans, and as to how much fuel a pound of eggs would furnish; the same, too, with all sorts of every-day foods. But they came across quite a number of articles entering into the dietaries of the foreign-born people which were new to them, and of these they were obliged to make special analyses. Also, they came across various inferior articles in common use, such as low-grade and cheap flour, which did not correspond in food-value to the high-grade articles, and here again special analyses were requisite. In none of the studies was it found practicable to make an accurate reckoning of the kitchen waste.

The method adopted for reckoning the food-values of various articles purchased in the markets was very pretty and

*Special Washington Correspondence of the Boston Transcript.

simple. A turnip, for example, of a given weight contains a certain amount of substance that goes to make muscle and blood. Also, it contains a certain quantity of another kind of substance which furnishes fuel for running the body-machine. A human being requires fuel as much as a locomotive does, else he would run down and come to a stop. Fat is a fuel; sugar and starch are fuels. You eat half a pound of sugar and it contributes nothing to make muscle and blood in your body, but to your body it is just what coal is to the locomotive. It is the same way with starch or with the fat of meats. If you want blood and muscle you must eat the foods which supply that sort of material, such as lean meat or peas and beans. Most foods contain both fuel-stuff and muscle-stuff, but the proportions vary. Accordingly, it is of the utmost importance to how much of each is contained in each kind of food.

Extraordinary difficulty was encountered in obtaining the consent of Italians for dietary studies in their homes. They did not see why the government should wish to inquire as to their private domestic arrangements; to weigh the flour and meat which they ate, and to write a report on the subject. There must be something unpleasant behind it, and, anyway, it was impertinent. Nevertheless the essential facts were secured by careful investigation. It appears that the chief articles of food of the Italians in American cities are wheat flour (or bread) macaroni and noodles. Potatoes, beans and peas also furnish an economical source of nutriment. People of this nationality, after acquiring residence in this country, cling to their native dietary habits with extraordinary persistence. They consume a great deal of macaroni, which, fortunately for them, is made in the United States. The same is not the case, however, with Italian oil, wine and cheese, which even the poorest families utilize daily, though they have to be imported and are proportionately expensive. Such articles are comparatively cheap in Italy, and so this transplanted population has become accustomed to their use.

One of the most interesting branches of the investigation described had relation to the Russian Jews. It appears that these Jews—at all events those of them who are orthodox—are extremely careful to adapt their diet to the requirements of ecclesiastical law, and the preparation of their food is equally governed by religious considerations. Of course the Jews in general have their rules about these matters, which are apt to be carefully observed, but among them there are no people more strict in this regard than the Russians. So far as vegetables and fruits are concerned, there is no prohibition against anything, but when it comes to meats very elaborate regulations must be observed. The animals must be slaughtered in a particular manner, all of the blood being removed from the body by severing arteries in the neck. In order to get rid of the last of the blood the meat is usually soaked in water for several hours after being bought, some of the nutritious constituents being thereby lost.

The orthodox Jews buy their chickens alive and kill and dress them according to their own customs. They seem to eat comparatively little fat. In general, among the families studied, the orthodox Jews were in better health than the unorthodox Jews, who are restricted by no religious rules to a prescribed manner of living. In most of the Jewish families studied the condition of the rooms was, to say the least, untidy; potato parings, bones and all other food-refuse were thrown upon the floor and swept up once a day. Such families always clean the house thoroughly once a year—i. e., at the time of the Passover. A less thorough cleaning is usual whenever a birthday in the family is celebrated.

There is no question of the fact that even the poorest people, though they waste very much less than the well-to-do, actually throw away a great deal that might be saved, simply because of ignorance of a few matters easily understood. It can not be expected of them that they should know what per cent. of a pound of beefsteak goes to make blood and muscle, and how much of it is fuel for the running of the body-machine. They go to the market, and they buy what seems most palatable at the smallest price. This is very well as far as it goes, but some of the most palatable articles of diet—as fruits, for example—are the least sustaining. Even this remark, however, is not to be made without reservation, inasmuch as fruits are desirable for health, and a few of them, like the banana, are very nutritious indeed.

The experts have a curious way of reckoning the fuel-power in a pound of food. They estimate it in terms of "calories." This is simple enough, when it is understood, inasmuch as a calory represents a certain amount of "go" for the machine, like a turn of the driving-wheel of a locomotive. So, when it is said that a man requires about 3,000 calories to run him for twenty-four hours, it is easily comprehended what is meant. The trouble with many people, and particularly very poor people, is that they select foods which contain too little fuel-stuff in proportion to the muscle-forming substance, or vice versa.

A special study was made of Bohemian families. It was ascertained that these families purchased their food at Bohemian markets, but that the character of the supplies was not peculiar. It is a custom at Bohemian markets, however, to give a piece of liver and a bone with each piece of beef sold—an item interesting to frugal housewives. Chopped beef and pork are common articles of food among the imported Bohemians. The butcher has a platter of chopped beef on one side of him and a platter of chopped pork on the other, and he mixes them to suit the individual customer. The milk dealers in these markets also sell skimmed milk and cream separately. Among the Bohemians the term "milk" is applied exclusively to skimmed milk. They usually buy more or less cream, which is added to the milk, the mixture being called "milk and cream." The amount of cream added depends upon the purse of the purchaser.

The dietaries of quite a number of Bohemian families were accurately studied, and it was found that the expenditure for food per individual averaged eleven cents a day. This result indicated a very wise and prudent expenditure for table purposes. It was found that in nearly all cases the Bohemians obtained their food at a lower cost than did the people of any other nationality studied. This was particularly noticeable in the animal foods, for which the price paid per pound was frequently not more than two-thirds that paid by other families with equal incomes.

A special investigation was made with the purpose of ascertaining the modifications in diet attributable to residence in this country. For the accomplishment of this object, families were selected which had been in the United States for different lengths of time. From the data obtained it appeared that a gradual change in diet followed removal of residence by Bohemians to the United States, and it is supposed that similar observations would apply to immigrants of other nationalities. When the Bohemians first arrive their diet naturally tends to conform itself to that to which they have been accustomed. They consume large amounts of rye flour, pork in considerable quantity, and comparatively little beef, with hardly any wheat or corn, and no variety of green vegetables and fruits worth mentioning. As they become more accustomed to the conditions existing here, they consume less pork and more beef, more wheat flour and wheat bread, and less rye flour, and a greater variety of vegetables and fruits. The second generation, native-born, adopts the American diet pure and simple.

Special attention was devoted to French-Canadians, but the only notable fact deduced seems to have been that these people are remarkably fond of pie. They spend nearly as much on pastry as they do on bread—say pastry and cake, which come in the same category. Nobody can deny that pastry and cake are go d foods, but at a given price they are only one-fourth as sustaining as bread. Recently there was a good deal of discussion as to the nutritive quality of wheat bread. It was asserted that the article had been greatly overestimated, and many things were much more digestible and sustaining than the so-called "staff of life." However, the fact back of all this nonsense is that bread, whether made of wheat, corn or rye, is an extremely valuable dietary substance. It is a fuel food typically, containing a great deal of starch, but, in addition, it has about twelve per cent. of the substance which makes muscle and blood. No reasonable person would suggest that a man ought to be able to live and be healthy on bread alone, but it furnishes none the less a most admirable basis of diet.

Naturally, the poor are limited in their choice of foods. It appears to be recognized by them pretty generally that wheat flour in bread, or otherwise prepared, furnishes the largest return for money expended. Strange it seems to be obliged to state that, through the practical working of domestic necessity, the poor of the great cities have arrived at conclusions not far different from those obtained by the scientific experts In other words, they have ascertained by trial the foods from which they can get the largest amount of nourishment and the utmost of working power. The experts confess that they can hardly suggest any way in which, in certain cases, the money available could be expended more profitably for nutrients. Given a possible expenditure of $6 per week for food, there are poor families, and many of them, which get as much out of that sum in the shape of nutritive supplies as the best scientific knowledge could furnish.

In general, the experts say, the cost of a diet may be diminished by consuming less fruit, less expensive cuts of meat, and fewer vegetables than are ordinarily eaten. Fruits add comparatively little to the food value of a diet, although they are useful for other reasons. The cheaper cuts of meat are as nutritious as the more costly cuts, and may be prepared in such a way as to be very palatable. Vegetable foods are essential to a well-regulated diet. Wheat flour in the form of bread or macaroni is one of the most nutritious, and at the same time one of the cheapest foods. Most vegetables do not contain much food-material, because their bulk is largely water; but they stimulate appetite and furnish bulk.

The problem of proper sustenance of the poor in large cities is continually assuming greater proportions. In many cases the income of a family is so small that the greater part of it must be expended on the necessary food. Not infrequently, through ignorance of the nutritive value of different foods, unwise selection, and improper cooking and serving, the actual value of the food for nourishment is much less than might have been obtained for the same money expenditure. Obviously, the power of a man to do work depends upon his nutrition; a well-fed man has strength of muscle and of brain, while a poorly nourished man has not. It is a very interesting matter in this connection to mention that, according to the conclusions of the Government experts, a woman requires only four-fifths of the food of a man, the work performed by both being equal. A boy from fourteen to sixteen years old requires four-fifths the food of a man. A girl of the same age requires seven-tenths the food of a man. A child ten to thirteen years old requires six-tenths the food of a man. A child six to nine years old requires one-half the food of a man. A child three to five years old requires four-tenths the food of a man, and a child under two years old requires three-tenths the food of a man.—*Rene Bache, in The Sanitarium.*

